# A novel anti-CD146 antibody specifically targets cancer cells by internalizing the molecule

**DOI:** 10.18632/oncotarget.22736

**Published:** 2017-11-28

**Authors:** Marie Nollet, Jimmy Stalin, Anaïs Moyon, Waël Traboulsi, Amel Essaadi, Stéphane Robert, Nausicaa Malissen, Richard Bachelier, Laurent Daniel, Alexandrine Foucault-Bertaud, Caroline Gaudy-Marqueste, Romaric Lacroix, Aurélie S Leroyer, Benjamin Guillet, Nathalie Bardin, Françoise Dignat-George, Marcel Blot-Chabaud

**Affiliations:** ^1^ INSERM UMR-S 1076, Aix-Marseille University, UFR Pharmacy, Marseille, France; ^2^ CERIMED, European Center of Research in Medical Imaging, Aix-Marseille University, Marseille, France; ^3^ Department of Dermatology, Timone Hospital, Assistance Publique des Hôpitaux de Marseille, Inserm UMR-S 911, Aix-Marseille University, Marseille, France; ^4^ Service d'anatomie Pathologique, Aix-Marseille University, Timone Hospital, Marseille, France

**Keywords:** antibody, CD146, cancer, PET, therapy

## Abstract

CD146 is an adhesion molecule present on many tumors (melanoma, kidney, pancreas, breast, ...). In addition, it has been shown to be expressed on vascular endothelial and smooth muscle cells. Generating an antibody able to specifically recognize CD146 in cancer cells (designated as tumor CD146), but not in normal cells, would thus be of major interest for targeting tumor CD146 without affecting the vascular system.

We thus generated antibodies against the extracellular domain of the molecule produced in cancer cells and selected an antibody that specifically recognizes tumor CD146. This antibody (TsCD146 mAb) was able to detect CD146-positive tumors in human biopsies and *in vivo*, by PET imaging, in a murine xenograft model. In addition, TsCD146 mAb antibody was able to specifically detect CD146-positive cancer microparticles in the plasma of patients. TsCD146 mAb displayed also therapeutic effects since it was able to reduce the growth of human CD146-positive cancer cells xenografted in nude mice. This effect was due to a decrease in the proliferation and an increase in the apoptosis of CD146-positive cancer cells after TsCD146-mediated internalization of the cell surface CD146.

Thus, TsCD146 mAb could be of major interest for diagnostic and therapeutic strategies against CD146-positive tumors in a context of personalized medicine.

## INTRODUCTION

CD146 (or MCAM, Mel-CAM, MUC18, Gicerin, S-endo1) is a transmembrane glycoprotein of about 110 kDa [1]. This adhesion molecule belongs to the immunoglobulin superfamily, displays five extracellular domains of the V-V-C2-C2-C2 type, a transmembrane domain and a short cytoplasmic domain [2]. Two different isoforms of the cell surface CD146 have been identified that differ by their cytoplasmic tail. The CD146 molecule was initially discovered in human melanoma cells [3] and was then described in many other tumors such as pancreas [4], kidney [5], prostate [6] or breast [7] cancer. In these cells, CD146 expression constitutes a marker of poor diagnosis and correlates with an aggressive and invasive phenotype. This is due to the involvement of the molecule in tumor growth and dissemination [8]. Thus, its expression was shown to induce epithelial-mesenchymal transition [9]. In a recent paper, CD146 was also identified as a markedly up-regulated surface receptor in chemoresistant small-cell lung cancer (SCLC) cell lines and in chemoresistant SCLC patient-derived xenografts compared to matched treatment-naïve tumors [10]. Likewise, Liang *et al.* demonstrated a novel function of MCAM in conferring tamoxifen resistance in breast cancer [11]. However, CD146 is also expressed on the different cells constituting the vessels where it displays important physiological functions [1]. Thus, our laboratory identified the molecule in the endothelium [12] and other reports described its expression on smooth muscle cells and pericytes [13, 14]. In endothelial cells, CD146 is essentially present at cell-cell junctions and is involved in vascular permeability and leucocytes transmigration during inflammation [12, 15].

In view of the deleterious role of CD146 in tumor development and dissemination, it would be of major interest to specifically target the tumor form of CD146 (designated as tumor CD146) without affecting CD146 present in normal cells, in particular vascular cells (designed as physiological CD146). Many studies evidenced structural and functional differences between molecules expressed on either cancer or normal cells. Thus, different levels of glycosylation or different conformational states have been reported for CD146 [16]. In view of these data, our working hypothesis was that tumor CD146 could display structural features that differ from those of physiological CD146. We have thus generated antibodies able to recognize the extracellular domain of CD146 and screened them for their ability to recognize CD146 expressed in cancer cells but not CD146 expressed in vascular cells. Of interest, one antibody, that we referred to as TsCD146 mAb (for Tumor specific anti-CD146 monoclonal antibody) displayed these properties. This antibody was thus further characterized in order to evaluate its potential interest for diagnostic and/or therapeutic applications.

## RESULTS

### Generation of a newly developed monoclonal anti-CD146 antibody specifically targeting tumor CD146

The recombinant extracellular domain of CD146 was expressed in mouse myeloma cells and used as immunogen to generate rat monoclonal antibodies. Hybridomas were screened for clones producing antibodies that i/ bound to the cell surface CD146 expressed on cancer cells, and ii/ did not bind to the cell surface CD146 expressed on the surface of endothelial and smooth muscle cells. The hybridoma clone TsCD146 mAb (IgG1 subtype) was selected based on these criteria and was further characterized.

### Characterization of TsCD146 mAb

**Figure 1:**
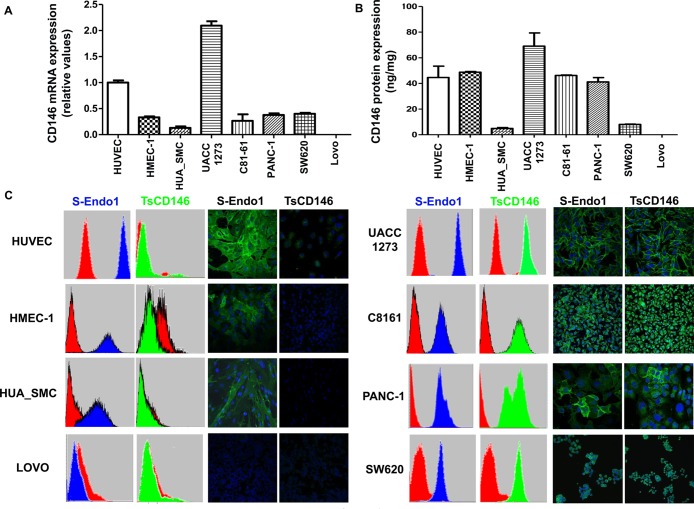
Specific detection of CD146 in cancer cells with TsCD146 antibody (**A**) CD146 mRNA expression was analyzed in the different cell lines by RT-PCR. The results are expressed as relative values compared to HUVEC. Results are the average of 3 independent experiments. (**B**) The protein expression of CD146 was analyzed in the different cell lines by ELISA assay. Results are expressed as relative values compared to HUVEC. Results are the average of 3 different experiments. (**C**) CD146 was detected in the different cell lines with the TsCD146 mAb and the control S-Endo1 antibody by flow cytometry and immunofluorescence. Control mouse and rat IgG were used and primary antibodies were revealed with a secondary antibody coupled to FITC. Images are representative of 3 different experiments.

Since it has been shown that many tumor types express CD146, we screened five cancer cell lines (two metastatic melanoma (UACC-1273 and C8161), a pancreatic (Panc-1) and two colonic (SW620 ad Lovo) cancer cell lines), with TsCD146 mAb and contrasted them with two types of micro and macro-vascular endothelial cells (HMEC-1 and HUVEC) and smooth muscle cells (HUA-SMC) used as a non-diseased control cell types. In these different cancer cell lines, only Lovo did not express CD146. CD146 expression was evaluated in these different cells at the mRNA level by RT-PCR with primers directed against the extracellular portion of CD146 and at the protein level by ELISA assay on cell lysates. In all cell types, except Lovo, we observed CD146 expression at the mRNA (Figure 1A) and protein (Figure 1B) levels. TsCD146 mAb was compared to the commercially available S-Endo1 antibody on its ability to bind to cancer cells, endothelial cells and smooth muscle cells. To this end, both flow cytometry and immunofluorescence experiments were performed (Figure 1C). Results show that the TsCD146 mAb was able to bind to UACC-1273, Panc-1, C81-61, SW620 cancer cells but not to Lovo cells which do not express CD146. It was not able to bind to HUVEC, HMEC-1 and HUA-SMC cells. In contrast, the S-Endo1 antibody was able to bind to all cells, except Lovo cells. In Panc-1 cells, the binding in immunofluorescence experiments appears very heterogeneous, both with S-Endo1 and TsCD146 mAbs, indicating that the Panc-1 cell line is probably composed of different cell types.

In addition, immunoprecipitation experiments were performed on the different cell lines with the TsCD146 mAb and revealed by western-blot. Results (Supplementary Figure 1) show that TsCD146 mAb immunoprecipitates CD146 only in UACC and Panc-1 cell lines, but not in HUVEC and HMEC-1 cells, and that the molecule migrated at approximatively 110 kDa.

### Use of TsCD146 mAb for immunodetection of cancer cells in biopsies of human tissues

**Figure 2:**
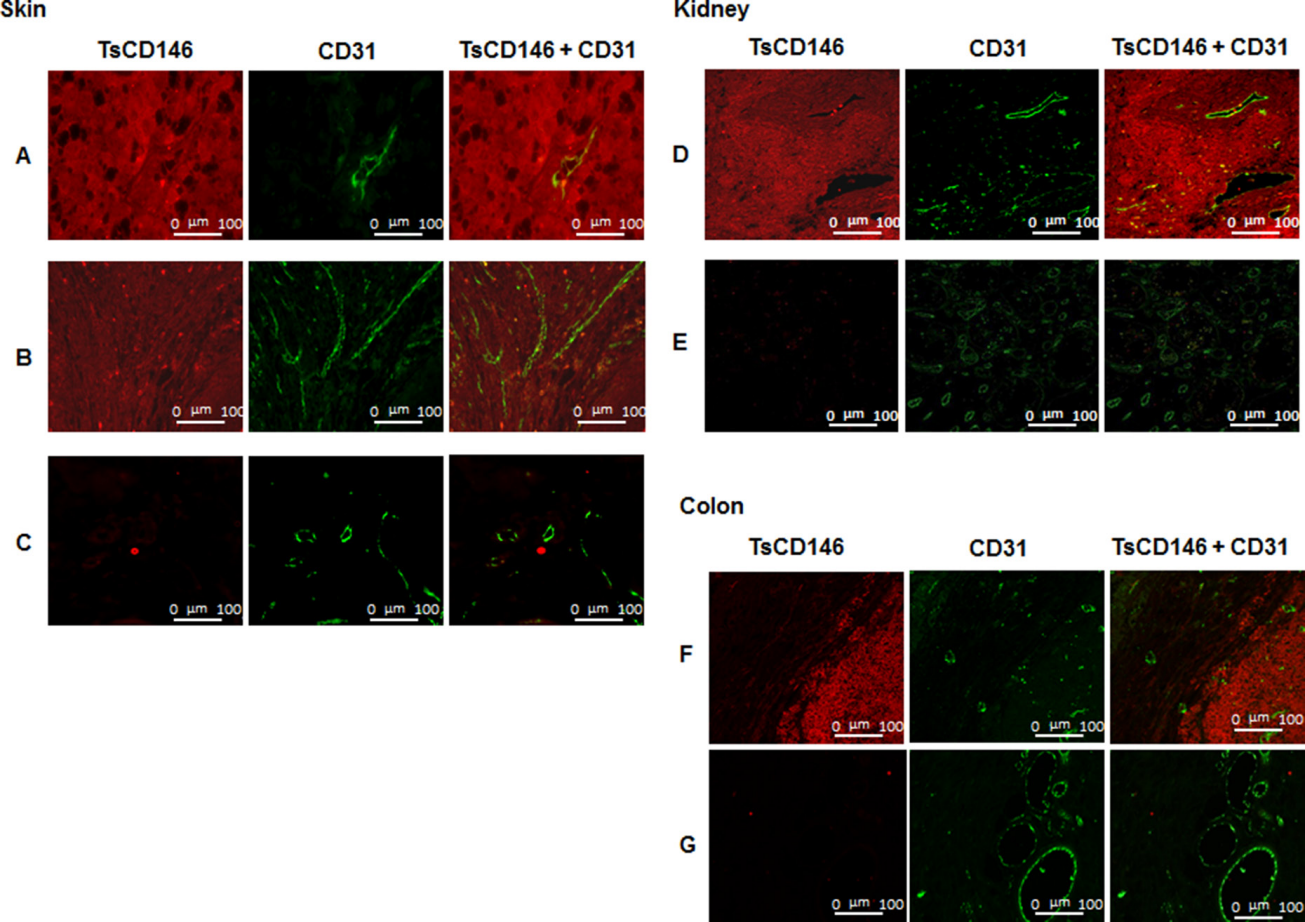
Immunodetection of CD146 in cancer cells with TsCD146 mAb in human biopsies Different biopsies of skin, kidney and colon were labeled with the TsCD146 mAb coupled to an Alexa 546 anti-rat antibody and analyzed by immunofluorescence. CD31 mAb coupled to an alexa 488 anti-rabbit antibody was also used and merge images are shown. For skin, human melanoma (**A**), verrucous carcinoma of skin (**B**) and normal skin (**C**) biopsies are shown. For kidney, renal carcinoma (**D**) and normal kidney (**E**) biopsies are shown. For colon, colonic adenocarcinoma (**F**) and normal colon (**G**) biopsies are shown. This experiment is representative of 3 experiments for each type of cancer. Bars correspond to 100 μm (Magnification 40X).

In order to demonstrate that TsCD146 mAb was able to detect CD146-positive tumor cells without binding to vascular cells, we carried out immunofluorescence experiments on human melanoma biopsies (Figure 2A) and verrucous skin carcinoma (Figure 2B) in comparison with normal skin biopsies (Figure 2C). Experiments were also performed on biopsies of renal carcinoma (Figure 2D) and colonic adenocarcinoma (Figure 2F) in comparison with normal kidney (Figure 2E) and normal colon (Figure 2G). Tissue sections were also labeled with an antibody directed against the vascular endothelium (anti-CD31 antibody). Binding of TsCD146 mAb was evidenced on tumor cells but not on endothelial cells which were however labeled with CD31 mAb as expected. No binding was observed with TsCD146 mAb on normal skin, kidney and colon. In contrast, S-Endo1 antibody was able to bind to both tumor and endothelial cells in renal carcinoma, as demonstrated by its colocalization with CD31 antibody in blood vessels (Supplementary Figure 2).

### Use of radiolabeled TsCD146 Fab'2 for immunodetection of melanoma cells by PET imaging

**Figure 3:**
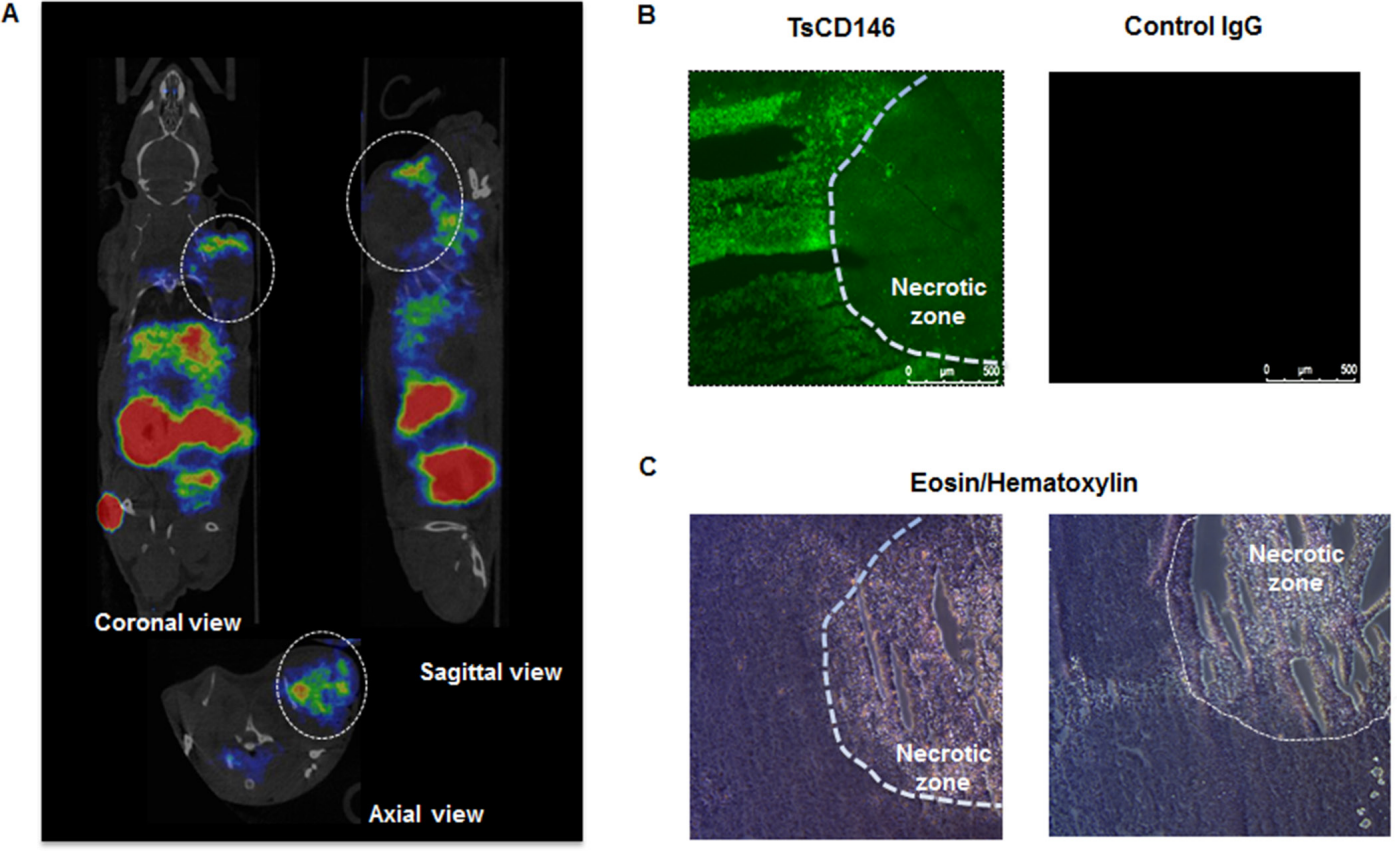
Immunodetection of CD146 with radiolabeled TsCD146 mAb in cancer cells xenografted in nude mice (**A**) Fab’2-TsCD146 was radiolabeled with ^68^Galium after NODAGA conjugation and injected in nude mice xenografted with C8161 melanoma cells. PET analysis showed that the tumor was detected with the radiolabeled antibody. Coronal, sagittal and axial views are shown. Tumors are located within dotted circles. Representative images of PET are shown. Experiments were performed with 6 different animals. (**B**) After sacrifice of the animals, tumors were labeled with TsCD146 mAb coupled to a FITC secondary antibody. A non-specific rat IgG was used as a control. The dotted line corresponds to the necrotic zone visualized in C. A representative image from 6 different experiments is shown. Magnification ×5. (**C**) After sacrifice of the animals, tumors were analyzed by histochemistry after treatment with eosin/hematoxylin. The dotted line corresponds to the necrotic zone. A representative image from 6 different animals is shown.

We investigated whether TsCD146 mAb was able to detect human melanoma cells *in vivo* using a xenograft model. To this end, we used Fab'2 fragment from TsCD146 mAb coupled to ^68^Ga (see Material and Methods). Nude mice were injected with C8161 cells and 36 days later with ^68^Ga Fab’2-TsCD146. Results obtained by PET imaging (Figure 3A) showed that the tumor could be visualized with the radiolabeled Fab’2. Of interest, only the external part of the tumor was labeled, without labeling in the central part. To further analyze this result, we performed immunofluorescence and histological experiments on these tumors after euthanizing animal. These *ex vivo* analyses confirmed the results obtained *in vivo*. Indeed, only the external part of the tumor was labeled with the TsCD146 mAb (Figure 3B). The inner part consisted of necrotic tissues (Figure 3C).

### Use of TsCD146 mAb for immunodetection of cancer cell microparticles in the plasma of patients with melanoma

**Figure 4:**
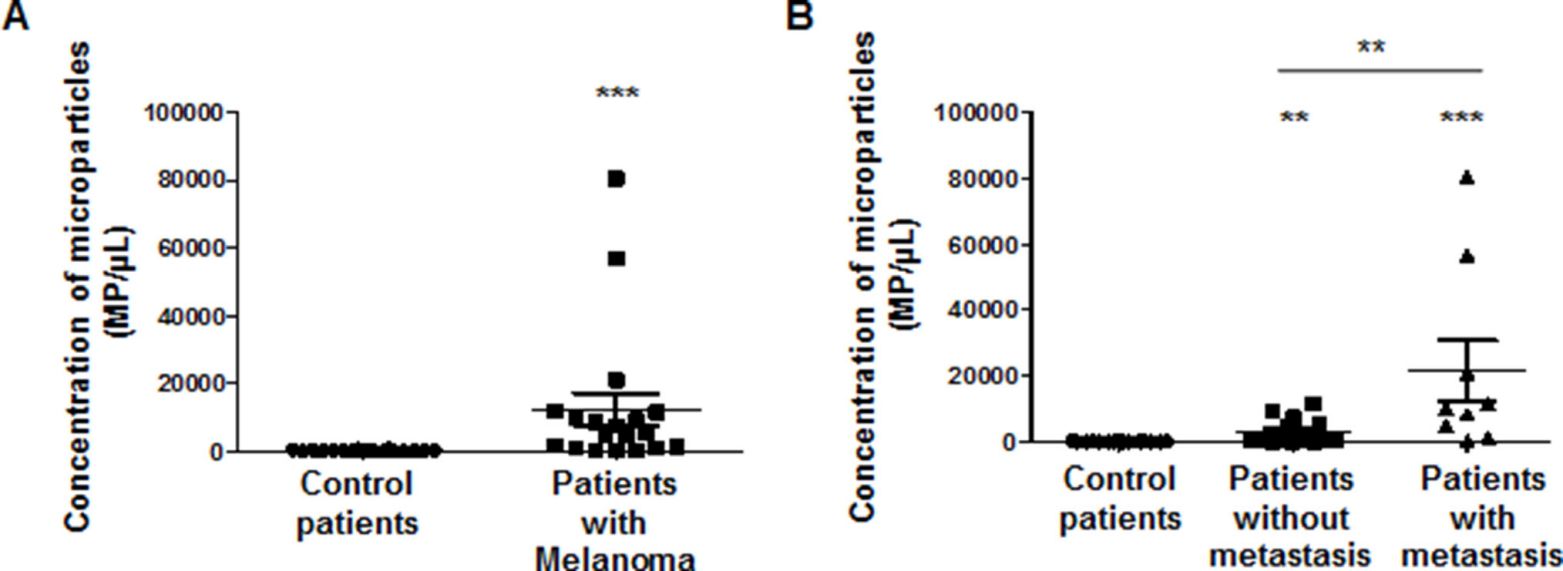
Immunodetection with TsCD146 mAb of CD146 expressed on microparticles in plasma samples from patients with melanoma Annexin V-positive/TsCD146-positive microparticles from plasmas of control patients and patients with melanoma at different disease stages were quantified. Control patients were compared to all melanoma patients (**A**) or to patients with melanoma at initial or metastatic stages (**B**). ^**^p < 0.01, ^***^p < 0.001 experimental versus control.

As cancer cells are able to generate microparticles which are secreted in the blood flow of patients and as microparticles carry many proteins from the mother cells, we investigated whether microparticles (MP) generated by melanoma cell lines or patients with melanoma were detected by the TsCD146 mAb. In a first series of experiments, MP were isolated from the culture medium of UACC melanoma cells and from the culture medium of HUVEC, and binding of TsCD146 mAb was analyzed together with the binding of the MP-specific marker annexinV. Supplementary Figure 3A shows that TsCD146 mAb did not bind to HUVEC MP but did bind to UACC MP. In view of these results, we performed a second series of experiments with plasmas from control patients and patients with melanoma (Supplementary Figure 3B). Altogether, results show that annexin V/Ts CD146-positive cancer MP were detected in the plasma of patients with melanoma whereas none were detected in the plasma of control patients. In addition, the number of annexin V/TsCD146-positive MP could be correlated with the initial *versus* metastatic stages of the tumor (Figure 4).

### TsCD146 mAb decreases proliferation and membrane expression of CD146 in cancer cells by internalizing the molecule

**Figure 5:**
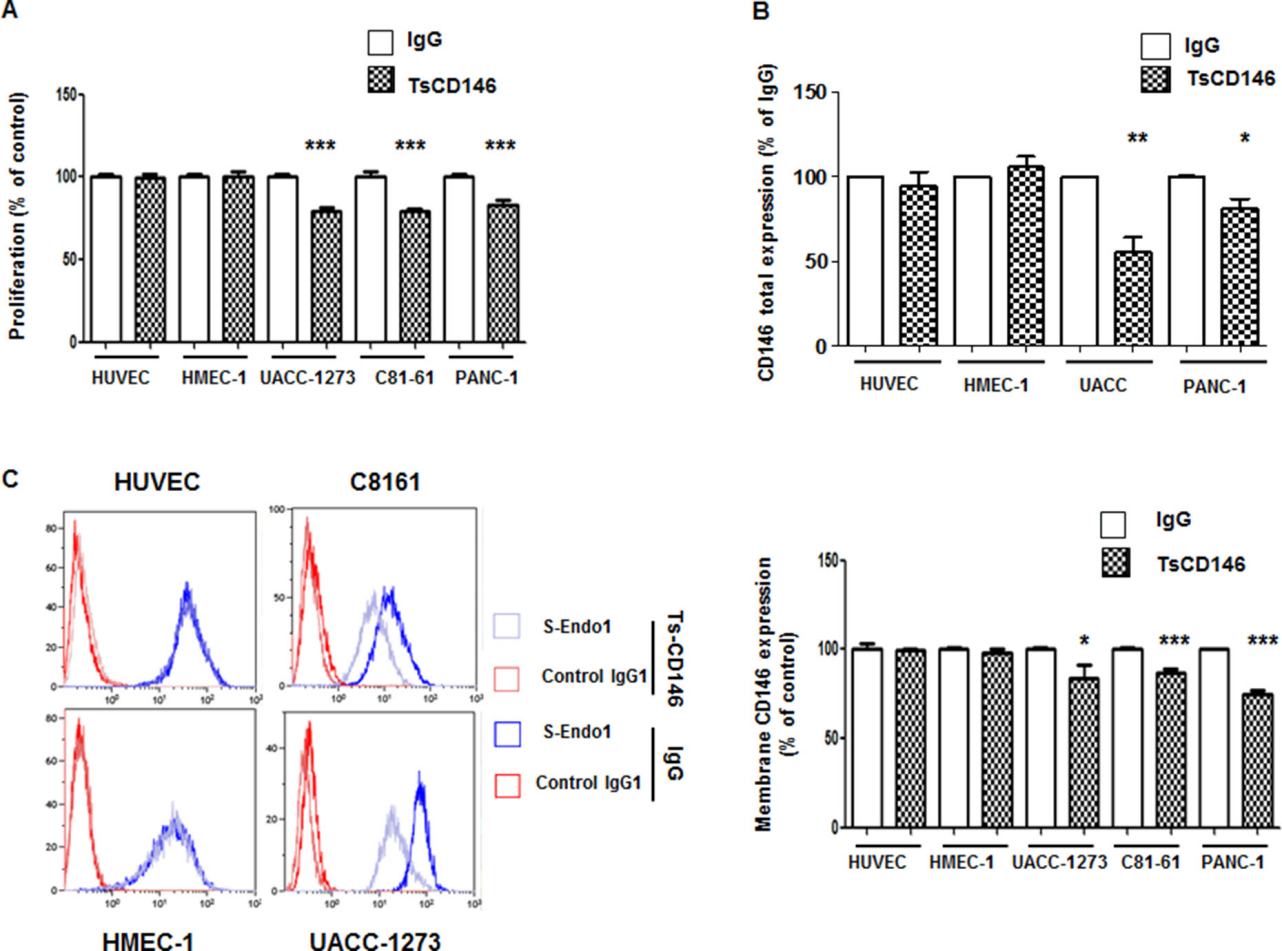
Effect of TsCD146 mAb antibody on cell proliferation and CD146 expression (**A**) Analysis of the proliferation of HUVEC, HMEC-1, UACC-1273, C81-61 and Panc-1 cells after 72 h in the presence of IgG or TsCD146 mAb (5 μg/ml). The results are expressed as mean values +/− standard deviation of 3 independent experiments. (**B**) Total CD146 expression was determined by ELISA on lysates of UACC, Panc-1, HUVEC and HMEC-1 cells after 72 h of treatment with control IgG or TsCD146 mAb. Mean values of three independent experiments are shown. (**C**) Membrane expression of CD146 was determined by flow cytometry with S-endo1 antibody in the different cell lines after treatment with either TsCD146 mAb (5 μg/ml) or IgG (5 μg/ml) for 72 hours. Representative FACS profiles are shown. The results are expressed as mean values +/− standard deviation of 3 experiments. ^*^p < 0.05, ^**^p < 0.01, ^***^p < 0.001, experimental versus control.

We tested the effect of TsCD146 mAb in cancer cell proliferation as compared to proliferation of endothelial cells. After a 72-hour treatment with the antibody at 5 μg/ml, we observed a 20–25% decrease in proliferation of UACC-1273, C81-61 and Panc-1 cells whereas there was no effect on HUVEC and HMEC-1 cells (Figure 5A).

Since an anti-proliferative effect of TsCD146 mAb was observed on cancer cells and since CD146 is involved in cell proliferation, we investigated whether this effect could be due to a decrease in the expression of CD146. In a first series of experiments, we analyzed the total expression of CD146 by ELISA on whole cell lysates. Results (Figure 5B) showed that total CD146 was reduced in cancer cells (by about 50% in UACC-1273 and 25% in Panc-1) but not in endothelial cells after 72 h of treatment with TsCD146 mAb. In a second series of experiments, we then investigated whether this could be due to a decrease in the membrane expression of CD146. The cell surface CD146 expression was thus evaluated by flow cytometry in cancer cells after 72 hours of treatment with the TsCD146 mAb or the control IgG. A significant decrease of about 20–25% in CD146 membrane expression was observed in the different cancer cell lines whereas there was no effect in endothelial cells (Figure 5C).

**Figure 6:**
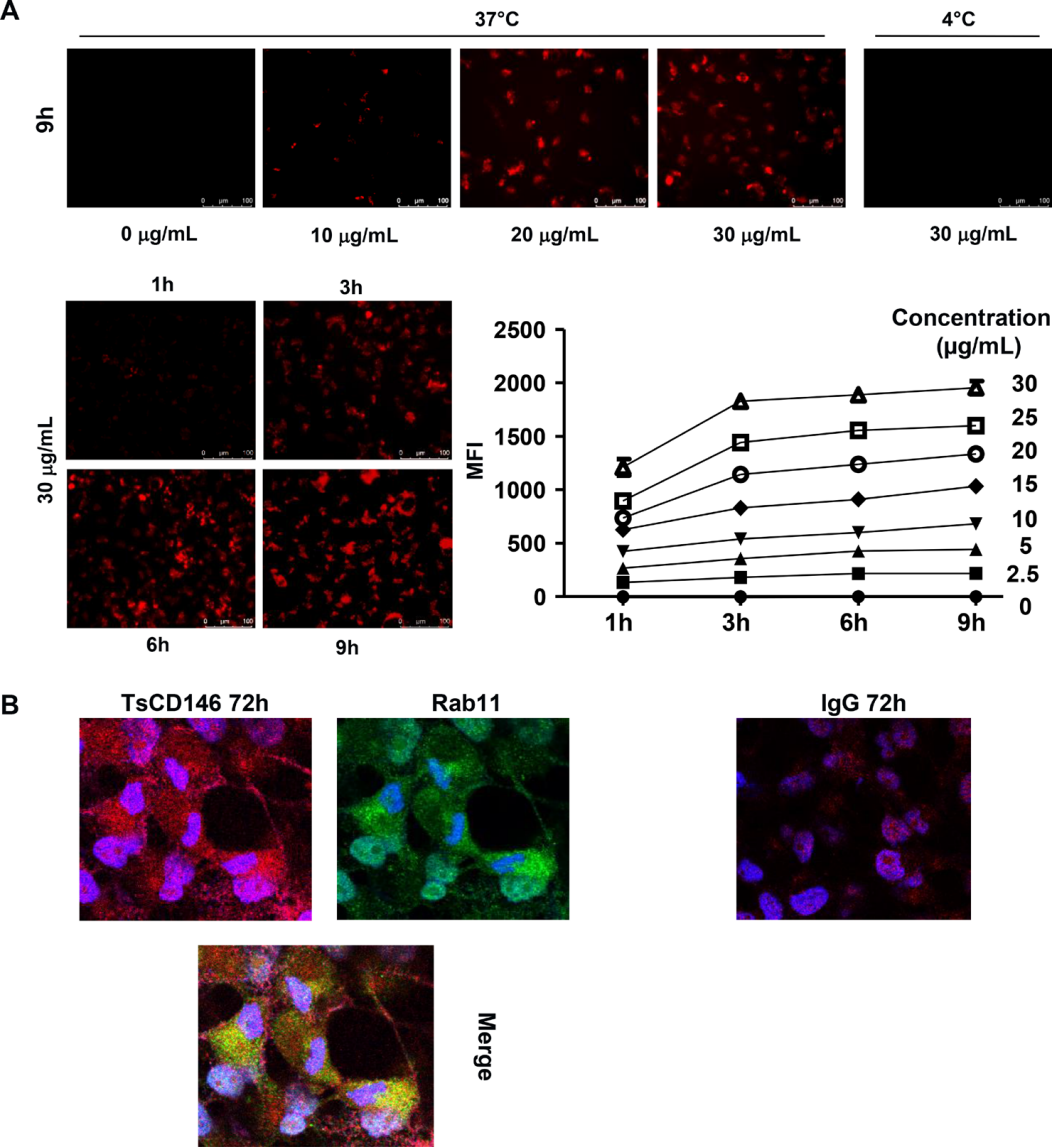
Effect of TsCD146 mAb antibody on CD146 internalization (**A**) Visualization and quantification of TsCD146 mAb internalization in C81-61 cells. C81-61 were seeded and incubated for 1 to 9 hours at 37°C with 2.5–30 μg/ml TsCD146 mAb conjugated to Protonex red 600 SE. Mean Fluorescence Intensity (MFI) of the signal was measured and visualized with a fluorescence microscope. As a control, experiments were also performed at 4°C for 9 hours with 30 μg/ml of the complex. The results are representative of 4 independent experiments. (**B**) Colocalization of CD146 and rab11 in C81-61 cells. After treatment of C81-61 cells with TsCD146 mAb for 72 h, cells were fixed and TsCD146 mAb was visualized together with rab11 by confocal microscopy. Merge image is shown. C81-61 cells treated for 72 h with control IgG are also shown. Nuclei are visualized in blue with DAPI. Images are representative of 3 independent experiments.

In view of these results, we performed experiments to determine whether this down-regulation of cell surface CD146 was due to cell surface CD146 internalization and degradation. To this end, we used a probe that becomes fluorescent in acidic subcellular compartments as endosomes or lysosomes. Results (Figure 6A) show that after treatment of C81-61 cells with the TsCD146 mAb, CD146 was directed towards intracellular acidic compartments of the cells at 37°C but not at 4°C, a decrease in temperature preventing the internalization of proteins from the membrane to the intracellular compartments. In addition, time-course experiments showed that internalization started rapidly since it could be observed as soon as three hours after the beginning of treatment with the TsCD146 mAb. Similar results were obtained with Panc-1 and UACC cells (Supplementary Figure 4). This result was confirmed by confocal microscopy experiments. Indeed, we showed that, after 72 h of treatment with TsCD146mAb, CD146 was colocalized with rab11 in intracellular compartments in C81-61 cells (Figure 6B).

### TsCD146 mAb induces apoptosis but not necrosis in cancer cells

**Figure 7:**
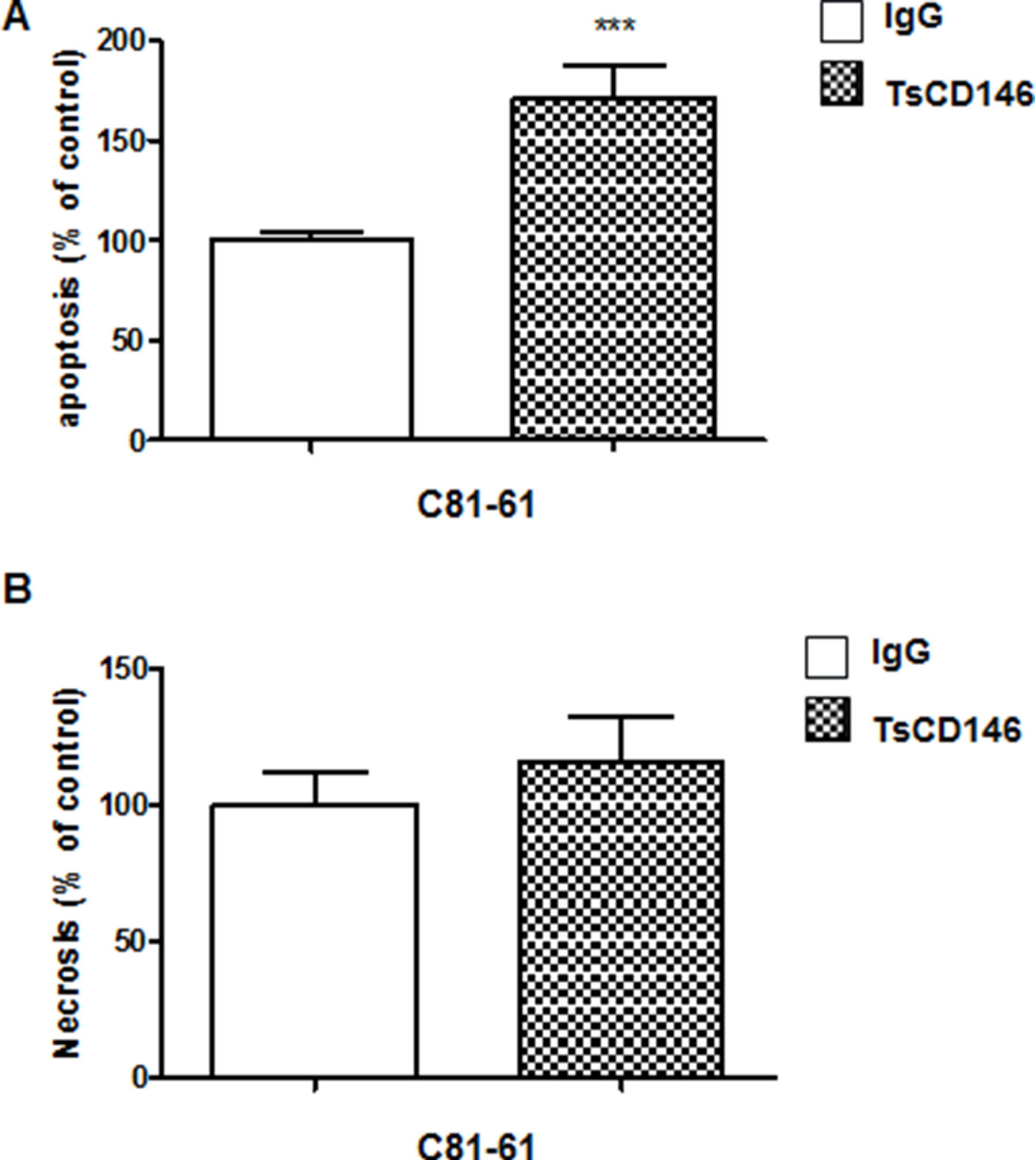
Effect of TsCD146 mAb antibody on cell apoptosis and necrosis in C81-61 cells (**A**) Analysis of apoptosis on C81-61 cells after 72 h in the presence of IgG or TsCD146 mAb (5 μg/ml). The results are expressed as mean values +/− standard deviation of 3 independent experiments. (**B**) Analysis of necrosis on C81-61 cells after 72 h in the presence of IgG or TsCD146 mAb (5 μg/ml). The results are expressed as mean values +/− standard deviation of 3 independent experiments. ^***^p < 0.001, experimental versus control.

We tested the effect of TsCD146 mAb in cancer cell apoptosis and necrosis. After a 72-hour treatment with the antibody at 5 μg/ml, we observed a 75% increase in apoptosis of UACC-1273 cells (Figure 7A) whereas there was no effect on necrosis (Figure 7B).

### TsCD146 mAb decreases tumor growth in animal models of xenograft

**Figure 8:**
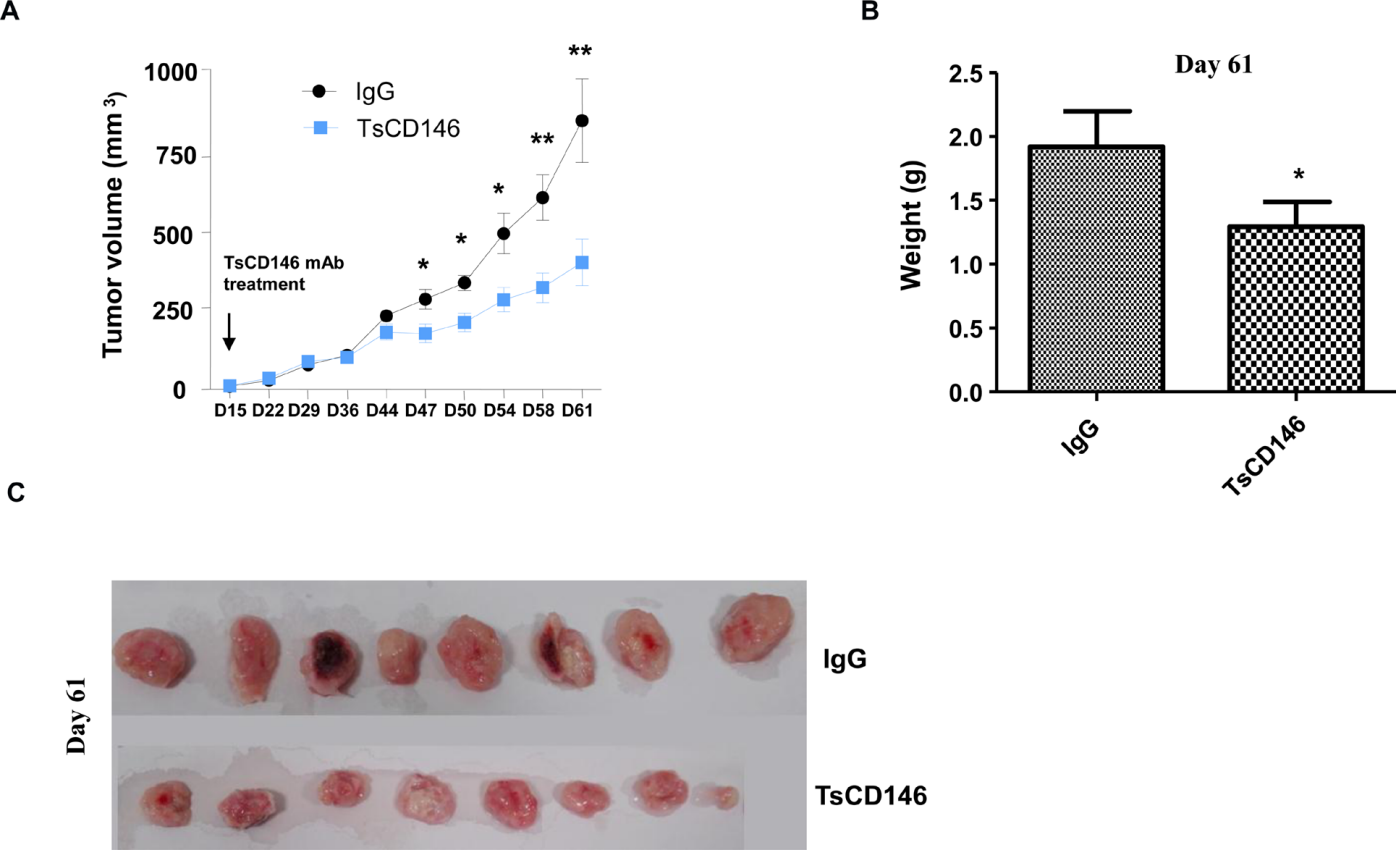
Effect of TsCD146 mAb on growth of C81-61 cells in an animal model of xenograft (**A**) 8 NOD/SCID mice xenografted with C8161 cells were treated for 46 days with control IgG or the TsCD146 mAb. Tumor volume was determined twice a week with a caliper. (**B**) Tumor weight was determined in IgG and TsCD146 mAb treated animals after euthanasia of the animals (day 61). (**C**) Tumors from IgG and TsCD146 mAb treated animals were imaged after euthanasia of the animals.

The effect of TsCD146 mAb was tested on tumor growth in C81-61 melanoma cells subcutaneously xenografted in NOD/SCID mice. When tumors reached about 20 mm^3^ (15 days), TsCD146 mAb or an isotype control IgG (rat IgG1 mAb) were injected twice a week over a 46 days period. Tumor growth was monitored by determination of relative tumor volume by caliper. Caliper measurement revealed a significant 50% decrease of tumor size in the group of mice injected with the TsCD146 mAb compared to the rat IgG control group after 46 days of treatment with the antibody (Figure 8A). Tumor weight was also determined at day 61 on isolated tumors after the sacrifice of animals and revealed that tumor weight was significantly reduced by about 40% in the TsCD146 mAb treated group as compared to the rat IgG control group (Figure 8B). At day 61, mice were euthanized and tumors were collected, weighted and photographed. The weight (Figure 8B) and size (Figure 8C) of tumors were significantly reduced in the TsCD146 mAb group, compared to the IgG group.

These results were confirmed in another model of CD146-positive tumors subcutaneously xenografted in nude mice, the Panc-1 cells. Using the same experimental set-up, we showed that tumor volume was significantly reduced by about 30% in the TsCD146 mAb treated tumor group as compared to the control group 46 days after the beginning of the treatment (Supplementary Figure 5).

## DISCUSSION

Early detection and treatment of cancer is a major public health issue. In order to efficiently fight the disease, personalized diagnosis and therapy are developed. This is based on the knowledge of the different proteins which are modified or involved in the development of the disease. Among them, CD146 has been described to be neo-expressed in numerous tumors and to be associated with a poor prognosis. Indeed, CD146-positive tumors are characterized by a high proliferation rate and a high capacity to disseminate. This is due, in particular, to the role of CD146 in epithelial-mesenchymal transition [14]. Many tumors neo-express CD146, in particular melanoma, prostatic, breast, lung, pancreatic, kidney, gastric and hepatic cancers [3–5, 7, 17–21]. However, CD146 is not only expressed in these cancer cells, but it is also physiologically expressed in different types of cells, primarily endothelial and smooth muscle cells constituting the vessels [8], and some activated T cells [22]. In endothelial cells, CD146 is of major importance since it is mostly expressed at the junction of the cells and is involved in vascular permeability, inflammation and angiogenesis [12, 15, 23]. In smooth muscle cells, CD146 is involved in the interaction with endothelium [1]. Finally, in Th17 cells, CD146 displays a major role in lymphocyte extravasation in the central nervous system [24]. Thus, both normal and cancer cells express CD146 at their surface. Likewise, microparticles contained in the bloodstream and generated from both endothelial [25] and CD146-positive cancer cells [26] exhibit CD146 at their surface. It appears to be very difficult to specifically target CD146 expressed in cancer cells or their derivatives (circulating cancer cells or microparticles), leading to major problems for diagnosis and/or targeted therapy. Specifically targeting the tumor form of CD146 with an antibody could thus allow detecting and targeting tumor cells without affecting vascular integrity and functions. Two neutralizing monoclonal antibodies targeting the membrane form of CD146 have been generated. ABX-MA1 decreases tumor growth and vascularization and targets both the tumor and the endothelial cell surface CD146 [27]. More recently, AA98 mAb, an antibody that targets the membrane-bound CD146 expressed by tumor vessels, has been generated [28]. This antibody presents a high anti-tumor activity. Finally, our team recently developed the M2J-1 mAb which specifically targets the soluble form of CD146 but not the tumor-specific membrane-bound form of CD146 [29]. Thus, currently, there is no antibody specifically targeting cancer cells available in the clinical setting.

Of interest, it has been frequently described that physiological and pathological protein isoforms can differ because of different sequences, post-translational modifications or three-dimensional conformations. Thus, many post-translational modifications of proteins occur in cancer cells such as glycosylation, acetylation, ubiquitination or sumoylation [30] that may explain different binding characteristics of antibodies. Likewise, different three-dimensional conformations can result from differences in oligomerisation. Thus, many differences could lead to different structural features of CD146 in cancer and normal cells. This prompted us to generate an antibody able to target tumor CD146 but not physiological CD146. Up to now, no structural difference between the two forms of CD146 has been described. It has only been reported that CD146 would be expressed as both monomeric and dimeric forms with differences between normal and cancer cells [31]. Thus, CD146 could be essentially expressed as a dimer in cancer cells and as a monomer in endothelial cells. Further studies will be necessary to test this hypothesis and a detailed characterization of the target of the TsCD146 mAb will be required to explain the specificity of the TsCD146 antibody used in the present study.

Regarding the interest in using TsCD146 mAb for diagnostic purposes, we have shown that it is able to detect CD146 in human biopsies of different tumors expressing CD146 and *in vivo* in tumor xenografts by PET imaging. Concerning this last technique, it could be useful for the specific detection of CD146-positive tumors before targeted therapy. Today, tumor cells are detected by imaging with common markers as ^18^FDG which do not permit to distinguish between tumor subtypes [32]. There is a need for new biomarkers able to discriminate between tumors that would pave the way for a personalized medicine. TsCD146 mAb could therefore constitute a biomarker of tumor dissemination. Of interest, we have shown that it is also able to detect microparticles from CD146-positive tumors in the plasma of patients with melanoma and that the number of CD146-positive microparticles was significantly increased in metastatic patients versus patients with initial tumors. Along this line, it will be of interest to test in future studies whether TsCD146 mAb is also able to detect circulating cancer cells that are released by tumors and also constitute biomarkers of tumor dissemination. In that way, this new antibody could thus constitute a new tool to detect CD146-positive tumors in liquid biopsies, a minimally invasive option of great interest in the clinical setting.

In addition to the diagnostic interest, our study shows that TsCD146 mAb could be of interest for therapeutic purposes. Indeed, our results demonstrate that treatment with this novel CD146 antibody induces a decrease in the expression of the tumor CD146 molecule at the membrane. This is due in part to an internalization of the molecule after treatment with the antibody, as demonstrated by the use of a pH-dependent fluorescent dye and the colocalization with rab11, a protein involved in vesicle transport between membrane and intracellular compartments [33]. This mechanism has been described for other antibodies, such as trastuzumab. This antibody is currently used for the treatment of breast cancer. Its mechanism of action involves internalization of the HER2 receptor, a marker of poor prognosis, as in the case of CD146 [34]. Thus, the tumor CD146 molecule could be removed from the plasma membrane and then be directed from the endosomal to the lysosomal compartments where it could be degraded. Further experiments will be necessary to further characterize this phenomenon. This reduction in CD146 membrane expression leads, at least in part, to a decrease in cell proliferation, as already reported [35], and could explain part of the observed therapeutic effect of the molecule. Another part could be due to tumor death through apoptosis since TsCD146 mAb induces a clear effect on cell apoptosis *in vitro*. Additional mechanisms as CDC or ADCC could also occur. This will have to be examined in further experiments. In xenograft cancer models, our experiments show that TsCD146 mAb administration leads to a significant decrease in tumor growth. In view of its mechanism of action, TsCD146 mAb could thus be of therapeutic interest, administrated either alone or coupled to biologically active cytotoxic payloads or drugs, in order to kill cancer cells after internalization. This second strategy has shown promising results recently [36]. In view of the present experiments, it is not possible to conclude whether the initially CD146-expressing xenografts persist or not as CD146 non-expressing tumors after treatment or whether cells that persist in mice are no longer able to bind the antibody through adaptative changes in the extracellular domain of the protein. Further studies will be necessary to address these questions. It will also be of interest to test other infusion methods of TsCD146 such as *i.v*. injection.

In conclusion, our study shows that it is possible to specifically target the tumor CD146 molecule. We have, for the first time, successfully generated a monoclonal antibody, referred to as TsCD146 mAb, that specifically binds to and internalizes tumor cell surface CD146. This antibody could be of interest for diagnosis since it is able to recognize CD146-positive tumors on biopsies and *in vivo* by PET imaging. It can also detect microparticles in the plasma of patients with CD146-positive tumors. In addition, because of its mechanism of action, this antibody could also be of interest for therapy since it is able to reduce the growth of murine CD146-positive tumor xenografts. It will now be of major interest to precisely define its specific epitope on cancer cells and to further study its mechanism of action before considering its use in human personalized medicine.

## MATERIALS AND METHODS

### Cell culture

HUVEC (Human Umbilical Vein Endothelial Cells) were grown in Endothelial Cell Growth Media (EGM-2 BulletkitTM) (Lonza, Amboise, France). The HMEC-1 (Human Microvascular Endothelial Cell Line) cell line was grown in Endothelial Basal Medium (EBM) (PAA, Velizy-Villacoublay, France) supplemented with 10% fetal calf serum (FCS), penicillin and streptomycin, 1%), L-glutamine (1%), epidermal growth factor (10 ng/mL) and hydrocortisone (50μg/mL). HUA-SMC (Smooth Muscle Cells) were grown in Dulbecco's Modified Eagles medium (DMEM) supplemented with 10% FCS.

Tumor cell lines PANC-1 (pancreatic cancer) and LOVO (colonic cancer) were cultured in DMEM (Life Technologies, Saint Aubin, France) supplemented with FCS (10%), PS (1%), L-glutamine (1%) and sodium pyruvate (1%). The tumor cell lines UACC-1273 (melanoma), C81-61 (melanoma) and SW620 (colonic cancer) were cultured in RPMI 1640 Glutamax™ (life technologies) supplemented with FCS (10%) and PS (1%).The cells were grown in a humidified atmosphere with 5% CO2 at 37°C.

### Generation of antibodies against CD146

Antibodies against CD146 were generated at the platform of monoclonal antibodies (Mi-mAbs; Marseille-Luminy, France) by injection in rats of a recombinant protein corresponding to the extracellular domain of CD146, generated in mouse myeloma. After obtaining the hybridoma, anti-CD146 antibodies were purified by affinity chromatography on a HiTrap protein G column (GE Healthcare). TsCD146 mAb was characterized as an IgG1 antibody.

### qPCR

Experiments were performed as previously described [29]. Primers were: GAPDH: R: 5′-GGTGGTCTCCTGACTTCAACA; F: 5′-GTTGCTG TAGCCAATTCGTTGT; CD146: R: 5′-GGCTAATGCCTC AGATCGATG; F: 5′-AATATGGTGTTGAATCTGTCTTG.

### Flow cytometry

Cells were processed as previously described [29] and data sorted by flow cytometry (GalliosTM Flow Cytometer, Beckman Coulter, Villepinte). The results were then analyzed using the Kaluza software (Kaluza^®^ Analysis Software, Beckman Coulter).

UACC derived microparticles were first isolated by serial centrifugations of UACC conditioned medium (3 centrifugations at 70,000 g for 1 h 30 each). MP were then labeled with 10 μl of AnnexinV-FITC (Tau Technologies, Netherlands) and 10 μl of various concentration of PE-TsCD146 mAb for 30 minutes at room temperature, in the dark. 500 μl of AnnexinV binding buffer was finally added and the sample analyzed by flow cytometry. A titration curve was established through determination of the maximum percentage of double positive AnnexinV/TsCD146 microparticles among AnnexinV-positive MP (data unshown). The vesicle suspension was also stained with an equivalent amount of isotopic control IgG to confirm staining specificity.

The same staining was realized on plasmatic MP derived from healthy patients or patients with melanoma. Briefly, PFP were prepared from EDTA blood collection tubes using a first centrifugation on ficoll sucrose gradient for 30 min at 800 g. Then the fraction above the PBMC ring was collected. Then 30 μl of PFP were thawed and stained with AnnexinV FITC and TsCD146 PE Ab or IgG isotypic control for 30 minutes at room temperature, in the dark. After addition of 500 μl of AnnexinV binding buffer + hirudine (Cryopep, Montpellier, France), 30 μl of MP count beads (Biocytex, Marseille, France) were added to evaluate the tumor MP concentration.

MP samples were analyzed on a Gallios Flow Cytometer using the Megamix+ FSC strategy previously published by our team [37]. Cancer MP were defined as AnnexinV /TsCD146-positive events.

### Confocal microscopy

Experiments were performed as previously described [29] with primary anti-CD146 antibodies S-endo1 (Biocytex) and TsCD146 mAb (dilution 1/200) and secondary anti-mouse and anti-rat (dilution 1/200) antibodies coupled to Alexa 488 diluted in PBS-saponin (0.2%) - FCS (10%) for 30 minutes. The coverslips were then mounted with DAPI (Prolong^®^ gold antifade reagent with DAPI, Invitrogen) mounting medium and observed with a confocal microscope (Leica SP5, Leica, Nanterre, France).

For experiments on biopsies, organs were fixed in 4% formalin and sectioned using a cryostat. The slides were then pretreated in a pH 6 citrate buffer at 96°C for 30 minutes. They were then preincubated for 30 minutes in PBS-BSA 0.5%-Triton 10× 0.1%. Sections were then incubated with primary S-endo1 and TsCD146 antibodies diluted 1/100 overnight in a humidity chamber then with anti-mouse and anti-rat secondary antibodies coupled to Alexa diluted 1/200 in PBS-BSA 0.5%-Triton 10× 0.1% for 1 hour. Samples were processed and imaged as described above.

For CD146-rab11 colocalization experiments, cells were first treated for 72 hours with TsCD146 mAb. Then cells were fixed with PFA 4% and incubated for 5 minutes in PBS-Triton 10X 0.2% followed by a 30 minutes incubation with PBS-BSA 0.5%. They were then incubated with rab11 (Abcam, Ab3612) and CD146 antibodies for 1 hour and secondary antibodies (anti-rat alexa 647 for TsCD146 mAb and anti-rabbit alexa 488 for rab 11) for 1 hour. Protein co-localization was assessed by confocal microscopy.

### CD146 ELISA

A capture enzyme-linked immunosorbent assay (ELISA) (Biocytex, Marseille, France), was used to determine the expression of CD146 as indicated by the manufacturer. All specimens were tested in triplicate.

### Immunoprecipitation and Western-blot experiments

Experiments were performed as previously described [29]. Briefly, immunoprecipitation of protein was performed by incubating 5 μg of antibody overnight in cell lysates. For these experiments, a standard buffer (150 mM NaCl, 20 mM Tris HCl (pH 7.5, 1 mM Na2EDTA, 1 mM EGTA, 1% Triton, 2.5 mM sodium pyrophosphate, 1 mM glycerophosphate, 1 mM Na3VO4, 1mg/ml leupeptin and 0.5 mM phenylmethylsulfonyl fluoride) was used (Cell Signaling). Protein A sepharose was then added in the lysate before centrifugation, and denaturation with NuPAGE sample-reducing agent (Invitrogen). Samples were then subjected to NuPAGE using 4–12% Novex Bis-Tris gels (Invitrogen) and separated proteins were transferred onto nitrocellulose membranes (Invitrogen). Protein was then revealed by western-blot. Samples were subjected to NuPAGE using 4–12% Novex Bis-Tris gels (Invitrogen) and proteins were transferred onto nitrocellulose membranes (Invitrogen). Membranes were probed with specific primary antibody (1/100) followed by secondary antibody coupled to peroxidase. Blots were revealed with the ECL substrate (Pierce).

### Proliferation experiments

Experiments were performed as previously described [29]. Briefly cells were seeded on 96-well plates (5.10^3^/well) and cultured in complete medium for 3 days. Cells were then treated for 72 h in completed medium with 5 μg/ml TsCD146 mAb. Cell proliferation was assayed by 5-bromo-2′-deoxy-uridine (BrdU) incorporation into cellular DNA using the BrdU Labeling and Detection Kit III (Roche Corporation). The absorbance was measured at 450 nm using a micro-plate reader (Safas, Monaco). Results were expressed as arbitrary units. Each experimental point was realized in triplicate.

### Internalization experiments with protonex red 600 SE

Cells were seeded in 96-well plates, 10,000 cells per well, in complete cell culture medium containing 2.5–30 μg/ml TsCD146mAb conjugated to Protonex red 600 SE (AAT Bioquest, CA). Protonex is a pH-sensitive dye that is non-fluorescent at basic pH (extracellular: culture medium) and fluorescent at acidic pH (intracellular: endosomes, lysosomes). Serial dilutions of antibodies were added and plates were incubated at 37°C for 3, 6 and 9 hours. Experiments were also performed at 4°C as a control in order to block the internalization processes. Mean fluorescent intensities (MFI) of intracellular Protonex were measured per well using Glomax (Promega).

### Analysis of apoptosis and necrosis

Apoptosis and necrosis of C81-61 cells were determined on C81-61 cells treated for 72 h with TsCD146 mAb 5 μg/ml or control IgG. Apoptosis/Necrosis detection kit ‘Abcam ab176750) was used as described by the manufacturer.

### Analysis of tumor growth in murine xenograft models

Xenografts of human tumor cell lines were produced by injecting tumor cells (5 × 10^6^ cells resuspended in PBS) subcutaneously in the back of NOD/SCID mice. When tumors reached 20 mm^3^, peri-tumoral administrations of purified TsCD146 mAb or rat control IgG were realized at a dose of 10 μg, twice a week, for 46 days. Tumor size was measured twice a week with a caliper and tumor volume was determined according to the equation: (length × width × thickness) × 0.5236. Tumor weight was also evaluated at the end of the experiment after euthanizing the animal.

### NODAGA conjugation and radiolabeling

TsCD146 Fab'2 were generated and purified with Amicon Ultra-0.5 3KDa Centrifugal Filter 500 μL (Millipore Corporation) and added 10 equivalents of p-NCS-benzyl-NODAGA in 0.2 M bicarbonate buffered. The mixture was left at 37°C for 3 h. The conjugate was then transferred to an Amicon Ultra-0.5 3 KDa Centrifugal Filter Devices 500 μL, concentrated in distilled water (0.5mg/mL) and stored at −80°C.

### Radiochemistry

Gallium was obtained in ^68^GaCl3 form using a commercial TiO_2_-based ^68^Ge/^68^Ga generator (Obninsk).^68^GaCl3 (200.69 ± 40.97 MBq/0.5 mL) was eluted from a ^68^Ge-^68^Ga generator using 0.1 N HCl, and 4M ammonium acetate buffer (pH 7.4) was added. This solution was then added to NODAGA-TsCD146 Fab'2 (15 μg); the final pH of the mixture was 6.0. The reaction mixture was stirred at room temperature for 15 min with manual shaking.

### Micro PET imaging

Mice bearing C81-61 xenografts (*n* = 6) were injected IV at day 36 post implementation with 5–10 MBq of ^68^Ga-NODAGA-TsCD146 Fab'2 under 2% isoflurane anesthesia. PET images were acquired 1h30 and 3H00 after ^68^Ga-NODAGA-TsCD146 Fab'2 IV injection with a MedisoNanoPET/CT under 2% isoflurane anesthesia.

### Ethics committee approval

Animal experiments met the guidelines of the 2010/63/EU directive of the European Parliament and were performed under a protocol approved by an Institutional Animal Care and Use Committee (Aix-Marseille University). The procedures described above were conducted under an institutional approved animal use protocol (Marseille Ethical Committee) and under the supervision of an authorized researcher (B. Guillet; n°13328).

The experiments on human were performed retrospectively on plasma samples from patients with melanoma. They were performed in accordance with the Helsinki declaration of 1975, revised in 1983. Informed consent of the patients was obtained and the study was approved by the institutional ethical committee according to local regulation.

### Statistical analysis and expression of results

The data were expressed as mean values ± SEM of the indicated number of experiments. Statistical analysis was performed with the Prism software (GraphPad Software Inc., San Diego, CA, USA). The variance between the different groups to be compared was estimated before statistical analysis. When comparing more than two groups, a non-parametric one-way ANOVA followed by a Dunn's multiple comparison test was used. A *P* value < 0.05 was considered to be significant. Significant differences between two groups were determined using the unpaired student's *t*-test or Mann-Whitney test. A *P* value < 0.05 was considered to be significant.

The sample size was chosen for each type of experiment using an a priori power analysis. No exclusion criteria were taken into account; all the values obtained during the experiments were used for both *in vitro* and *in vivo* studies. In animal studies, the groups’ sizes were chosen using an a priori power analysis, the investigator blinded to treatment vs control groups and mice were distributed randomly between groups.

## SUPPLEMENTARY MATERIALS FIGURES



## Abbreviations

sCD146: soluble CD146
PET: positron emission tomography
FACS: Fluorescence-activated cell sorting
TsCD146: tumor specific CD146
mAb: monoclonal antibody
FCS: fetal calf serum
