# Supplementary material for: A novel anti-CD146 antibody specifically targets cancer cells by internalizing the molecule
Source: Oncotarget. 2017 Nov 28;8(68):112283–96. doi: 10.18632/oncotarget.22736 (PMC5762510; doi:10.18632/oncotarget.22736)
Supplement: Supplementary file 1 [file oncotarget-08-112283-s001.pdf]

A novel anti-CD146 antibody specifically targets cancer cells by internalizing the molecule

SUPPLEMENTARY MATERIALS

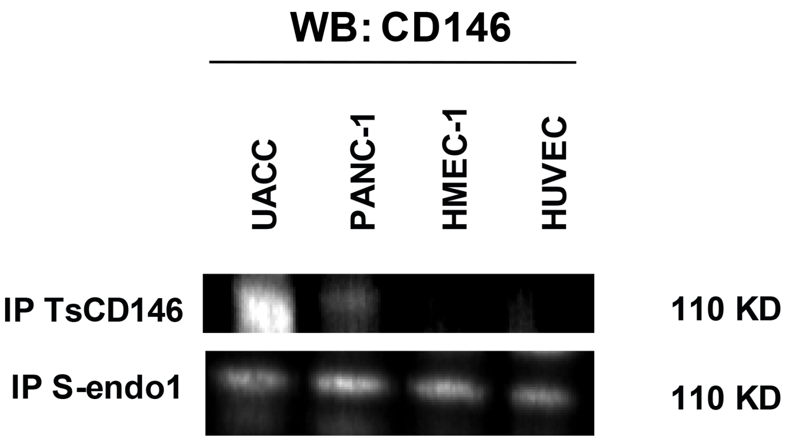

Supplementary Figure 1: Immunoprecipitation of CD146 in endothelial and cancer cells with TsCD146 mAb. CD146 was immunoprecipitated in lysates of UACC, Panc-1, HUVEC and HMEC-1 with TsCD146 or S-Endo1 mAbs and revealed by western-blot with COM7A4 antibody. The molecular weight of the protein is shown. The picture is representative of three independent experiments.

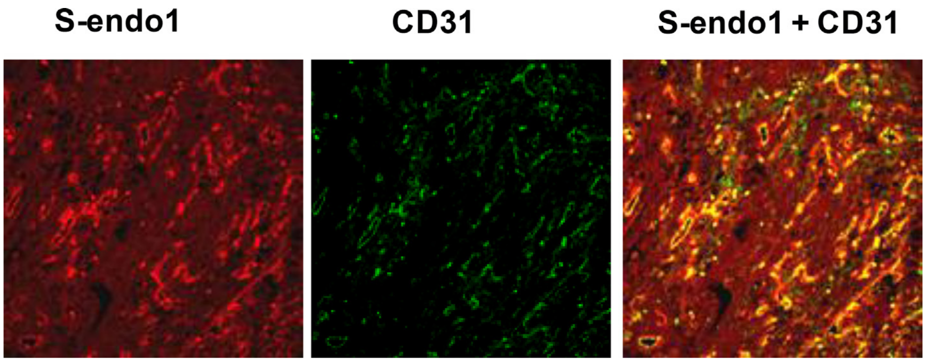

Supplementary Figure 2: Immunodetection of CD146 in cancer cells with S-endo1 mAb in human biopsies. Human renal carcinoma biopsies were labeled with the S-endo1 mAb coupled to an Alexa 546 anti-mouse antibody and analyzed by immunofluorescence. CD31 mAb coupled to an alexa 488 anti-rabbit antibody was also used and merge pictures are given. This experiment is representative of 3 independent experiments (magnification 40x).

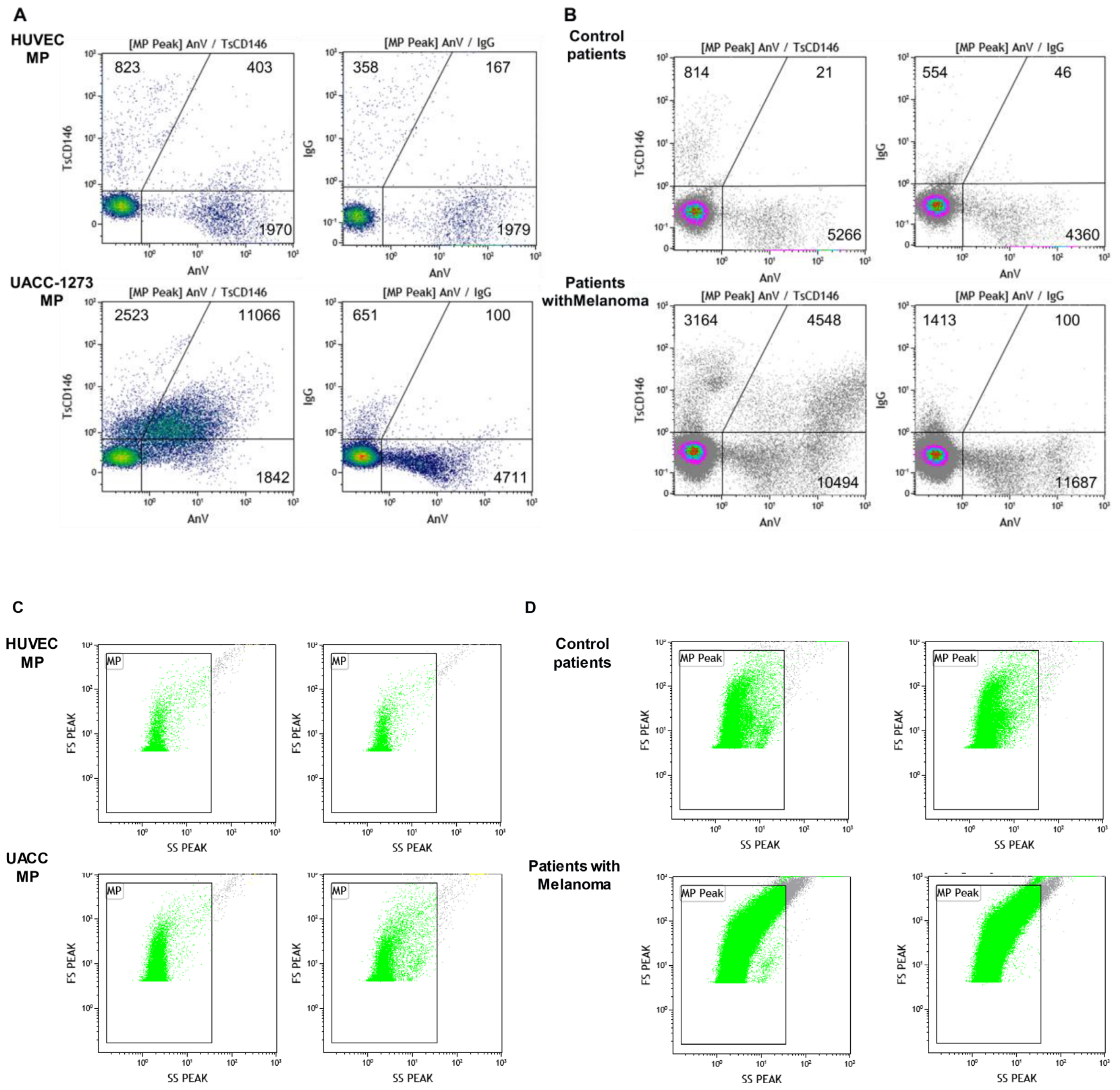

Supplementary Figure 3: Immunodetection with TsCD146 mAb of CD146 in microparticles secreted by cancer and endothelial cells and in plasma samples from control patients and patients with melanoma. (**A**) Annexin V-positive microparticles from HUVEC and UACC-1273 cells were analyzed by flow cytometry with TsCD146 mAb-PE and IgG1-PE control antibody. Images are representative of at least 4 different experiments. (**B**) Annexin V-positive microparticles from plasma samples from control patients or patient with melanoma were analyzed by flow cytometry with TsCD146 mAb-PE. Experiments were also performed with control IgG1-PE. (**C**–**D**) Side scatter and forward scatter are given for MP derived from cells in culture (C) and plasma samples from patients (D).

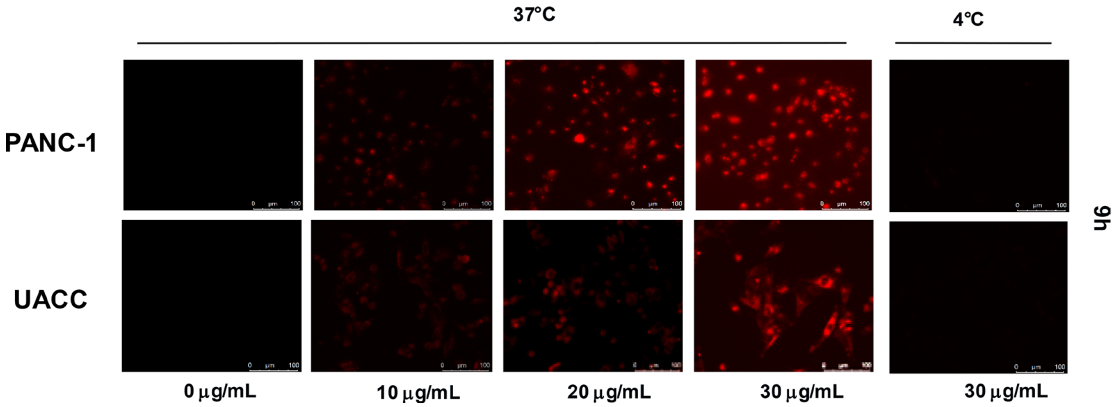

Supplementary Figure 4: Internalization of TsCD146 mAb in Panc-1 cells. Visualization of TsCD146 mAb internalization in Panc-1 and UACC cells. Cells were seeded and incubated for 1 to 9 hours at 37°C with 10–30 µg/ml TsCD146 mAb conjugated to Protonex red 600 SE. Fluorescence intensity of the signal was visualized with a fluorescence microscope. As a control, experiments were also performed at 4°C for 9 hours with 30 µg/ml of the complex. The results are representative of 4 independent experiments.

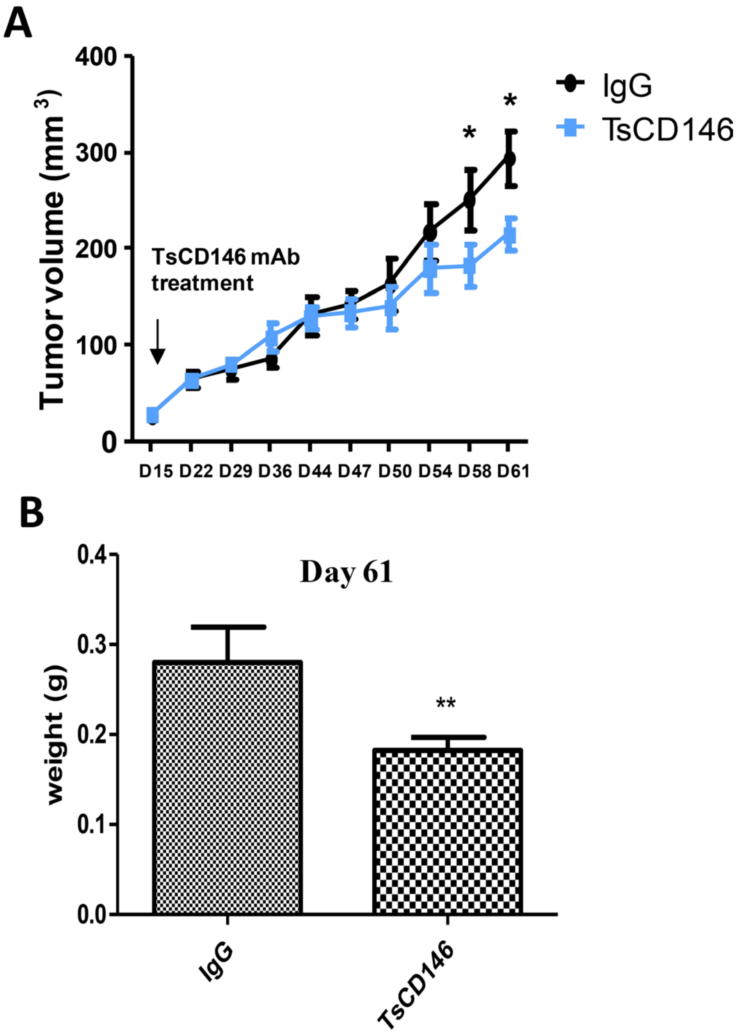

Supplementary Figure 5: Effect of TsCD146 mAb on growth of Panc-1 cells in an animal model of xenograft. 8 NOD/SCID mice xenografted with Panc-1 cells were treated for 46 days with control IgG or the TsCD146 mAb. Tumor volume was determined with a caliper (**A**) and tumor weight was measured at day 61, after euthanasia of the animals (**B**). Mean values ± SEM are given. *,***p* < 0.05, *p* < 0.01, experimental versus control.
